# The Regulation of Endothelin-1 in Pregnancies Complicated by Gestational Diabetes: Uncovering the Vascular Effects of Insulin

**DOI:** 10.3390/biomedicines11102660

**Published:** 2023-09-28

**Authors:** Bianca R. Fato, Sally Beard, Natalie K. Binder, Natasha Pritchard, Tu’uhevaha J. Kaitu’u-Lino, Natasha de Alwis, Natalie J. Hannan

**Affiliations:** 1 Therapeutics Discovery and Vascular Function in Pregnancy Group, Mercy Hospital for Women, University of Melbourne, Heidelberg, VIC 3084, Australia; bfato@student.unimelb.edu.au (B.R.F.); sally.beard@unimelb.edu.au (S.B.); nkbinder@unimelb.edu.au (N.K.B.); natasha.dealwis@unimelb.edu.au (N.d.A.); 2 Department of Obstetrics and Gynaecology, Mercy Hospital for Women, University of Melbourne, Heidelberg, VIC 3084, Australia; natashalpritchard@gmail.com (N.P.); t.klino@unimelb.edu.au (T.J.K.-L.); 3 Diagnostic Discovery and Reverse Translation in Pregnancy Group, Mercy Hospital for Women, University of Melbourne, Heidelberg, VIC 3084, Australia

**Keywords:** endothelin-1, gestational diabetes mellitus, insulin, endothelial dysfunction, pregnancy complications, vascular reactivity

## Abstract

Gestational diabetes mellitus (GDM) is a condition of pregnancy defined by new-onset hyperglycemia. GDM is associated with impaired maternal endothelial and vascular reactivity. Endothelin-1 (ET-1) is a potent vasoconstrictor that contributes to endothelial dysfunction, however, its abundance and actions in GDM are unclear. Maternal plasma was obtained from pregnancies complicated by GDM (*n* = 24) and gestation-matched controls (*n* = 42); circulating ET-1 levels were assessed by ELISA. Human omental arteries from healthy pregnancies and those complicated by GDM were dissected from omental fat biopsies and collected at cesarean section. mRNA expression of ET-1 and its receptors, ET_A_ and ET_B_, in addition to vascular cell adhesion molecule-1 (VCAM1) and intercellular adhesion molecule-1 (ICAM1) were assessed by qPCR (*n* = 28). Using wire myography, we investigated vascular constriction to ET-1 (10^−11^–10^−4^ M) in omental arteries from pregnancies complicated by GDM, compared to gestation-matched controls (*n* = 7). GDM cases were stratified by clinical management, diet intervention (*n* = 5), or insulin treatment (*n* = 6). Additionally, arteries from healthy pregnancies were treated with insulin (1 mU/mL (*n* = 7) and 10 mU/mL (*n* = 5)) or vehicle control. Vasoactive response to ET-1 was measured via wire myography. Circulating ET-1 levels and mRNA expression of the ET-1 system in omental arteries were not found to be significantly different between pregnancies complicated by GDM compared to healthy controls. However, we found insulin treatment during pregnancy and in ex vivo models reduced ET-1 vasoconstriction of maternal vasculature in GDM. These data suggest insulin may improve vascular function in GDM, however, further investigation is needed to define the role of ET-1 in pregnancy.

## 1. Introduction

Gestational diabetes mellitus (GDM) affects around 16.5% of pregnancies worldwide [1]. Key to the pathology of GDM is chronic insulin resistance during pregnancy, presenting clinically as spontaneous hyperglycemia. To manage the hyperglycemic state underpinning GDM, patients receive treatment via lifestyle interventions, such as diet (low glucose diet) and exercise, or if insufficient, drug therapies, such as insulin or metformin, to regulate their blood glucose levels. However, the effectiveness of these lifestyle interventions and drug therapies in alleviating the long-term cardiovascular impacts of this complication and the increased risk of developing Type 2 diabetes after pregnancy is not known [2,3,4,5,6].

Secondary to the significantly elevated blood glucose levels in GDM, the maternal vasculature is damaged by the hyperglycemic state and the milieu of chronic inflammation that leads to endothelial and vascular dysfunction. The endothelium (inner cellular layer of vasculature) plays a significant role in regulating vascular homeostasis and tone. Due to its contribution to vascular tone, dysfunction of the endothelium can lead to widespread vascular dysfunction and sustained aberrant constriction. Changes in the function, structure, and integrity of the endothelium during pregnancy are known to be associated with GDM [7,8]. The endothelin-1 (ET-1) pathway is a key mediator implicated in endothelial dysfunction, however, its regulation in the vasculature of pregnancies complicated by GDM remains unclear.

**Figure 1:**
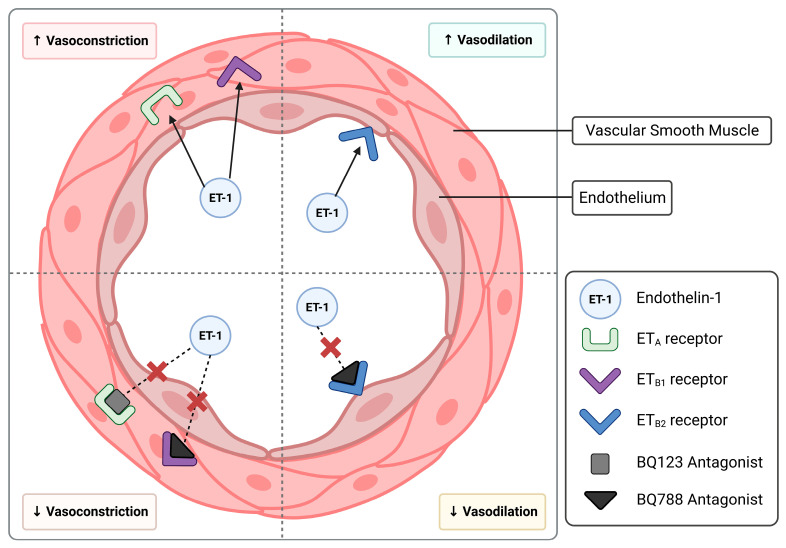
Schematic diagram of the endothelin-1 system, including its vasoconstrictive and dilatory actions on arteries. This figure highlights the antagonism of the endothelin-1 receptors, ET_A_ and ET_B_, using BQ123 and BQ788 antagonists, respectively (indicated by the red X).

Human endothelin is a 21-amino-acid polypeptide that is constrained by two intrachain disulfide bridges. The potent vasoconstrictor, ET-1, is predominantly produced by endothelial cells and is known to be a significant contributor to and feature of endothelial dysfunction. ET-1 acts through its two receptors, ET_A_ and ET_B_ (Figure 1), which are differentially distributed across the endothelial and vascular smooth muscle cells of blood vessels [9]. ET_A_ is predominantly expressed in the vascular smooth muscle cells, and its activation directly induces vasoconstriction [10]. ET_B_ is expressed on both the endothelium and vascular smooth muscle cells, and its activation can induce constriction or relaxation of blood vessels [11]. ET_B2_ receptors predominantly promote smooth muscle contraction and hence constriction of blood vessels, and conversely, ET_B1_ receptors located on the endothelium stimulate vasodilation through the release of nitric oxide (NO) and prostacyclin [12]. The activation of the ET_A_ and ET_B_ receptors has been explored previously with known receptor antagonists, BQ123 and BQ788, respectively [13]. In addition to stimulating constriction of vessels, ET-1 stimulates cytokine production and secretion of reactive oxygen species [14], implicating its contribution to a pro-inflammatory vascular environment prevalent in GDM [15].

During an uncomplicated pregnancy, circulating ET-1 levels typically remain low and decrease throughout gestation [16]. Studies investigating ET-1 levels in pregnancies complicated by GDM show mixed results. Several studies have reported elevated ET-1 in the maternal circulation of pregnancies complicated by GDM, particularly in pregnancies that also feature hypertension or develop concurrent preeclampsia [16,17]. Due to its vasoconstrictor actions, elevated ET-1 levels could contribute to the vascular pathology of GDM and potentially be a therapeutic target. However, when hypertensive disorders of pregnancy are excluded from analysis, there is no significant difference in the concentration of ET-1 in pregnancies complicated by GDM [18].

Here, for the first time, we aimed to investigate the regulation of the ET-1 system and its potential role in vascular dysfunction in pregnancies complicated by GDM. We used primary human samples collected from healthy and GDM pregnancies in both in vitro and ex vivo investigations. Importantly, we investigated whether there are differences in this system with the chosen management and obstetric care, including lifestyle dietary intervention or insulin treatment (in cases where dietary changes alone are not sufficient) to regulate blood sugar. Changes in vascular response or potential vascular benefits based on the chosen management strategy (diet or insulin) remain largely unknown.

## 2. Materials and Methods

### 2.1. Tissue Collection

This study was approved by the Mercy Health Human Research Ethics Committee (HREC/R11-34 and HREC/R14-11). Prior to sample collection, informed, written consent was obtained from participating patients attending Mercy Hospital for Women (Heidelberg, Victoria).

Term (delivery 37–41 weeks’ gestation) human placentas (*n* = 5) were collected from healthy, normotensive pregnancies (in 2021). Placental tissue was collected within 30 min of cesarean delivery. Tissue was cut from four sites of the placenta and washed in cold phosphate-buffered saline (PBS; 137 mM NaCl, 10 mM Na_2_NPO_4_, 1.8 mM KH_2_PO_4_, 2.7 mM KCl, pH 7.4) for explant dissection.

Maternal whole blood samples were collected at term (refer to Table 1 for patient characteristics and gestation at time of collection) from patients with pregnancies complicated by GDM (treated with either diet or insulin) and healthy gestation-matched controls (in 2021–2022). GDM was diagnosed according to the Australasian Diabetes in Pregnancy Society guidelines [19], where an oral glucose tolerance test was conducted at 24–28 weeks of gestation, and patients had either a fasting venous plasma concentration of ≥5.5 mmol/L glucose and/or ≥8.0 mmol/L glucose 2 h after a 75 g oral glucose load. Patients were given a diet intervention treatment when their fasting glucose readings were maintained below 5.5 mmol/L over a 2-week period post-diagnosis. However, patients with fasting glucose readings greater than 5.5 mmol/L were prescribed insulin for optimal glucose control. Subsequently, whole blood was collected at term delivery in 9 mL ethylenediaminetetraacetic acid (EDTA) tubes and subsequently centrifuged at 1500× *g* for 10 min. The plasma fraction was collected and stored at −80 °C until further assessment.

Omental fat biopsies collected from pregnancies complicated by GDM (treated with either diet or insulin) and healthy gestation-matched controls (in 2022–2023) were stored in Ca^2+^-free Krebs physiological salt solution (NaCl 120 mM, KCl 5 mM, MgSO_4_ 1.2 mM, KH_2_PO_4_ 1.2 mM, NaHCO_3_ 25 mM, D-glucose 11.1 mM) overnight (16 ± 2 h) at 4 °C to wash out anesthetics from surgery. Omental arteries were dissected and used in wire myograph experiments as described below; patient characteristics of omental samples are detailed in Table 2.

### 2.2. Enzyme-Linked Immunosorbent Assay (ELISA)

The concentration of circulating ET-1 was measured in maternal plasma collected from patients diagnosed with GDM who were treated with diet (*n* = 12) or insulin (*n* = 12) and normotensive pregnant gestation-matched control patients (*n* = 42). Plasma levels were measured using the Human Endothelin-1 ELISA Kit (R&D systems, Minneapolis, MN, USA), according to the manufacturer’s instructions.

### 2.3. Assessment of Vascular Reactivity

Vascular assessment was conducted as described previously [20,21]. In brief, human omental arteries were dissected from surrounding connective tissue and mounted on the 620 M Wire Myograph (Danish Myo Technology, Hinnerup, Denmark). A Krebs salt solution (heated to 37 °C) filled the chambers, which were continuously bubbled with carbogen (95% oxygen, 5% carbon dioxide). Using the DMT normalization module on LabChart software (ADInstruments, Bella Vista, NSW, Australia), arteries were normalization to 100 mmHg pressure (13.3 kPa, IC/IC100 = 1). The vascular reactivity of each vessel was assessed as previously described [20]. Using high potassium physiological salt solution (KPSS), vascular smooth muscle reactivity was assessed, and endothelial integrity was assessed using the endothelial-dependent vasodilator, bradykinin (Sapphire Bioscience, Redfern, NSW, Australia).

### 2.4. ET-1 Induced Omental Artery Constriction in Omental Arteries from Healthy Pregnancies and Those Complicated by GDM

Following confirmation of artery integrity as described previously [20,21], omental arteries from healthy pregnancies (*n* = 7) and those complicated by GDM (treated with either diet, *n* = 5, or insulin, *n* = 6) were constricted with recombinant human ET-1. Cumulative half-log doses of ET-1 (10^−11^ M to 10^−7^ M; Abcam, Cambridge, United Kingdom) were added every 2–3 min, until a plateau was reached; vascular response was measured with each addition of ET-1. Changes in constriction were normalized to the pre-determined maximum constriction induced by 50 mM KPSS.

### 2.5. Effect of ET-1 Receptor Antagonists on ET-1 Induced Omental Artery Constriction

The effects of ET_A_ receptor antagonist BQ123 and ET_B_ receptor antagonist BQ788 were assessed on omental arteries collected from pregnancies complicated by GDM (treated with insulin, *n* = 5) and healthy gestation-matched controls (*n* = 3–8). To investigate differences in the function of ET_A_ and ET_B_ receptors in GDM, following confirmation of artery integrity [20,21], arteries were incubated in BQ123 (1 µM; Sigma-Aldrich, St Louis, MO, USA), BQ788 (5 µM; Sigma-Aldrich, St Louis, MO, USA), a combination of BQ123 (1 µM) and BQ788 (5 µM), or vehicle control (ethanol) prior to constriction with recombinant ET-1 (10^−11^ M to 10^−7^ M). Following treatment, the antagonist or vehicle control treatment remained in the bath with ET-1 (concentrations as detailed above). Changes in measured constriction were normalized to the pre-determined maximum constriction induced by KPSS.

### 2.6. Effect of Insulin Treatment on ET-1 Induced Constriction

The effect of insulin treatment on ET-1-induced constriction was measured in omental arteries collected from healthy term pregnancies (*n* = 5) by wire myography. Following confirmation of artery integrity, arteries were incubated in 1 or 10 mU/mL of ActRapid Insulin (McFarlane Medical and Scientific, Surrey Hills, VIC, Australia) or vehicle control (water) for 30 min. Following this incubation, arteries were constricted using recombinant ET-1 (10^−11^ M to 10^−7^ M; Abcam). Log doses of ET-1 were added approximately every 2–3 min or until a plateau was reached; vascular response was measured with each addition of ET-1. Changes in constriction were normalized to the pre-determined maximum constriction induced by KPSS.

### 2.7. Human Umbilical Vein Endothelial Cell (HUVEC) Isolation and Culture

Primary HUVECs were isolated as previously described [22,23]. In brief, the umbilical cord vein from normal term placentas was cannulated, and fetal blood was washed out. Pre-warmed 1 mg/mL collagenase solution (Worthington, Lakewood, NJ, USA) was infused into the cord, followed by incubation at 37 °C for 8 min to allow HUVECs to dissociate. HUVECs were recovered by pelleting and resuspension, followed by culture, and used between passages 1 and 3. Cells were seeded for treatment in M199 media (Life Technologies, Carlsbad, CA, USA) containing 10% fetal calf serum (Sigma-Aldrich, St Louis, MO, USA), 1% endothelial cell growth factor, 1% heparin (Sigma), and 1% Antibiotic-Antimycotic (AA, ThermoFisher Scientific, Waltham, MA, USA) and incubated under 20% O_2_, 5% CO_2_ at 37 °C overnight.

### 2.8. In Vitro Treatment with Insulin and ET-1 Receptor Antagonists

Isolated primary HUVECs were treated with insulin and/or ET-1 receptor antagonists (BQ123 and BQ788) in fresh media. The dose of insulin and ET-1 receptor antagonists used was matched to the vascular studies performed. Using a model established previously [22], primary HUVECs were pre-treated for 2 h with 10 ng/mL tumor necrosis factor-α (TNF-α; Life Technologies), a pro-inflammatory cytokine, to induce endothelial dysfunction. Following pre-treatment, cells were then incubated (20% O_2_, 5% CO_2_ at 37 °C) for a further 24 h with 1 or 10 mU/mL of Act Rapid Insulin, and ET-1 receptor antagonists, BQ123 (1 µM) and/or BQ788 (5 µM) in the presence of TNF-α. Cell lysates were collected for RNA extraction.

### 2.9. MTS Cell Viability Assay

Cell viability was assessed following treatment using the MTS assay, CellTiter 96-Aqueous One Solution (Promega, Madison, WI, USA), according to the manufacturer’s instructions. Optical density was measured using a Bio-Rad X-Mark Microplate Spectrophotometer (Hercules, CA, USA) and BioRad Microplate Manager 6 software. The assays demonstrated that treatment with insulin or ET-1 receptor antagonists did not affect the cell viability of HUVECs.

### 2.10. Quantitative Real-Time Polymerase Chain Reaction (qPCR)

We collected HUVECs and human omental arteries from pregnancies complicated by GDM (treated with either diet, *n* = 5, or insulin, *n* = 7) in addition to gestation-matched healthy control arteries (normotensive, *n* = 18). Using the GenElute^TM^ Mammalian Total RNA Miniprep Kit (Sigma), RNA was extracted according to the manufacturer’s instructions. We quantified the RNA concentration using a Nanodrop 2000 spectrophotometer (ThermoFisher Scientific, Scoresby, VIC, Australia). Using the Applied Biosystems High-Capacity cDNA Reverse Transcription Kit (Thermofisher), RNA was converted to cDNA according to the manufacturer’s instructions on the iCycler iQ5 (Bio-Rad, Hercules, CA, USA).

Quantitative Taqman PCR was performed to quantify RNA expression of Endothelin-1 (*EDN1*, Hs00174961_m1), ET_A_ (*EDNRA*, Hs03988672_m1), ET_B_ (*EDNRB*, Hs00240747_m1), Vascular Cell Adhesion Molecule 1 (*VCAM1*; Hs01003372_m1), and Intercellular Adhesion Molecular 1 (*ICAM1*; Hs00164932_m1), as well as reference genes *YHWAZ* (Hs01122454_m1), *B2M* (Hs99999907_m1), and *Actin* (Hs99999903_m1). All primers were purchased through Life Technologies. Taqman qPCR was performed on the CFX384 (Bio-Rad) with the following run conditions: 50 °C for 2 min, 95 °C for 10 min, 95 °C for 15 s, 60 °C for 1 min (40 cycles). All data were normalized to the reference gene as an internal control and calibrated against the average Ct of the control samples; data shown as the fold change Geomean (+/−95% CI) and the y-axis on a log scale. All cDNA samples were run in duplicate.

### 2.11. Collection and Culture of Placental Explants

Placental explants were collected as described previously [22,23]. Placental explants were cultured in 6-well plates (40–60 mg per well), in Gibco™ M199 supplemented with 5% fetal calf serum and 1% AA. Explant tissue was cultured at 37 °C, 8% O_2_ (normoxic conditions), and 5% CO_2_ for 48 h under standard glucose (5 M) and high glucose (25 M) levels. Following this, conditioned media was collected and slowly frozen for 48 h before transfer to storage at −80 °C for subsequent myograph experiments. Media (standard and high glucose concentrations) without placental explants were exposed to the same culture conditions and duration for use as controls in myograph experiments.

### 2.12. Effect of Conditioned Placental Explant Media on ET-1 Induced Constriction

To investigate the effect of placental explant conditioned media, prior to constriction induced by recombinant ET-1 (10^−11^ M to 10^−7^ M; Abcam), healthy term omental arteries (*n* = 7) were incubated with 50% placental conditioned media (in Krebs) for 1 h. The four treatments were as follows; standard glucose placental conditioned media, high glucose placental conditioned media, and control standard and high glucose media that did not culture placental tissue. Once incubated, doses of ET-1 were added approximately every 2–3 min or until a plateau was reached; the vascular response was measured with each addition of ET-1. Changes in constriction were normalized to the pre-determined maximum constriction induced by KPSS.

### 2.13. Statistical Analysis

Maternal circulating ET-1 (ELISA) and mRNA expression of the ET-1 pathway (qPCR) data were assessed for normal distribution, and a one-way ANOVA was used to test the differences between the three groups (GDM diet, GDM insulin, or Control).

Myograph constriction curves were produced using non-linear regression (log[agonist] vs. response—four parameters or [agonist] vs. response—four parameters, as appropriate). Differences between responses to the agonist at each concentration were tested for significance using mixed-effects analysis with Šidák correction for multiple comparisons. The analysis of the area under the curve (AUC), maximum constriction, and (log)EC50 were tested for normal distribution and statistically tested as appropriate; the data were tested with an unpaired (parametric) or Mann–Whitney *t*-test.

*p*-values < 0.05 were considered significantly different, and all data are expressed as mean ± SEM. Statistical analysis was performed using GraphPad Prism 8.4.3 software (La Jolla, CA, USA).

## 3. Results

### 3.1. Circulating ET-1 Levels and Vascular ET-1 mRNA Expression Is Not Altered in Pregnancies Complicated by GDM Compared to Healthy Gestation-Matched Controls

**Figure 2:**
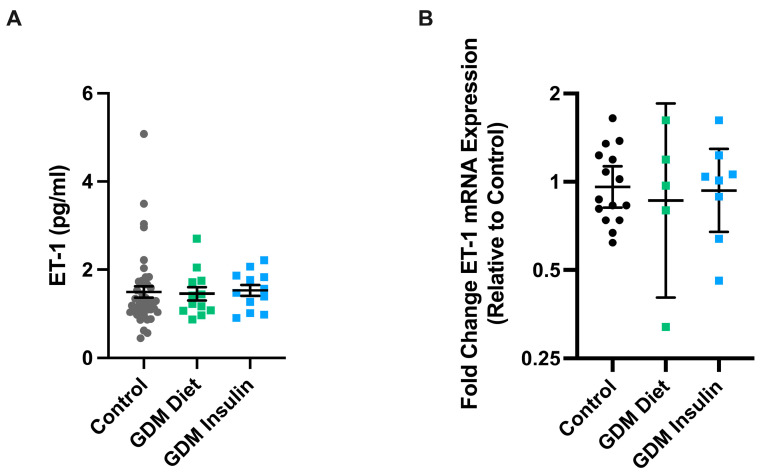
Maternal circulating ET-1 levels and vascular mRNA expression are similar at term in pregnancies complicated by GDM, compared to healthy controls. Circulating ET-1 concentrations were measured by ELISA in samples of plasma (**A**) from patients with pregnancies complicated by GDM who were treated with diet (*n* = 12, green squares) or insulin (*n* = 12, blue squares) and from uncomplicated pregnancies at matched gestation controls (*n* = 42, dark grey circles). The mRNA expression of ET-1 was measured in omental arteries (**B**) collected from pregnancies complicated by GDM who were diagnosed and subsequently treated with either diet (*n* = 5) or insulin (*n* = 8), compared to gestation-matched healthy term controls (*n* = 15). Individual points represent individual patients, and the data is expressed as mean ± SEM for ELISA data, and qPCR expressed as the log fold change of 2^−ΔΔCT^ Geometric (95% CI) relative to control.

We first assessed whether levels of the potent vasoconstrictor, ET-1, were altered in the maternal circulation or vasculature of pregnancies complicated by GDM (diet and insulin-treated). No significant differences in the levels of ET-1 were observed in the plasma of pregnancies complicated by GDM treated with either diet (*n* = 12) or insulin (*n* = 12) compared to healthy gestation-matched controls (*n* = 42) (Figure 2A). Similarly, we found no significant difference in the mRNA expression of ET-1 in maternal omental arteries collected from GDM pregnancies treated with diet (*n* = 5) or insulin (*n* = 8) compared to gestation-matched controls (*n* = 15) (Figure 2B).

### 3.2. Omental Arteries Collected from Pregnancies Complicated by GDM and Treated with Insulin Demonstrated Reduced Constriction Compared to Arteries from Pregnancies Managed with Diet or Healthy Controls

**Figure 3:**
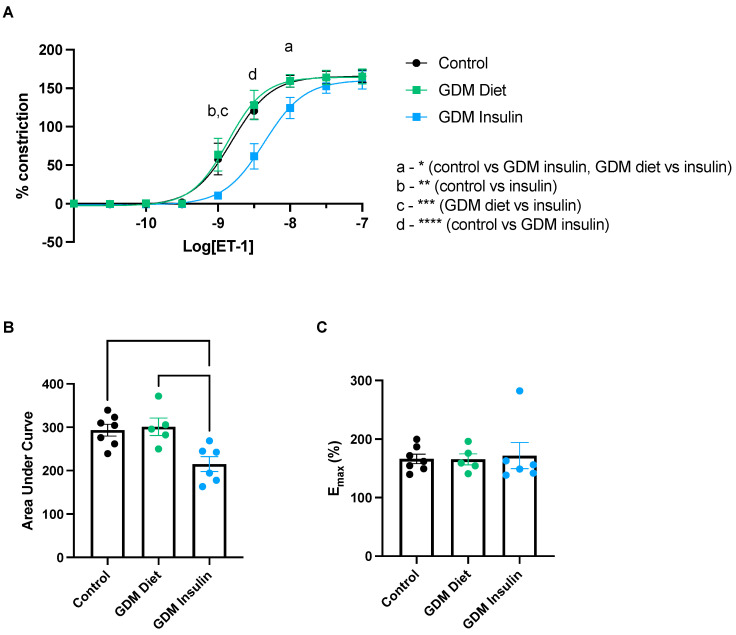
Omental arteries collected from pregnancies complicated by gestational diabetes mellitus (GDM) and treated with insulin constrict less in response to endothelin-1 (ET−1). Constriction curves (**A**), analysis of the area under the curve (**B**), and maximum constriction (**C**) generated by increasing logarithmic doses of a known vasoconstrictor, ET-1, in omental arteries collected from pregnancies complicated by GDM, insulin- (blue circles) and diet-treated (green circles), and from uncomplicated pregnancies at matched gestation (normal controls, black circles). The percentage of constriction was normalized to the maximum constriction induced by 50 mM potassium salt solution (KPSS). Individual points represent individual patients (*n* = 5–7). The data is expressed as mean ± SEM. Significance is indicated as follows: * *p* < 0.05, ** *p* < 0.01, *** *p* < 0.001 and **** *p* < 0.0001.

We next assessed whether the vascular response to ET-1 is altered in pregnancies complicated by GDM. ET-1 induced constriction in omental arteries collected from GDM patients treated with insulin was significantly reduced compared to omental arteries from diet-treated GDM patients or healthy controls; at dose 10^−9^ (normal vs. GDM insulin and GDM Diet vs. GDM insulin, *p* = 0.001), 10^−8.5^ (normal vs. GDM insulin and GDM Diet vs. GDM insulin, *p* < 0.0001), and 10^−8^ (normal vs. GDM insulin, *p* = 0.022; and GDM Diet vs. GDM insulin, *p* = 0.035) of ET-1 (Figure 3A). Analysis of the area under the curve (corresponding to total response over all doses of ET-1 assessed; Figure 3B) further demonstrated the total ET-1 induced constriction in omental arteries from GDM patients treated with insulin was significantly less than from patients with GDM treated with diet (*p* = 0.009), and healthy controls (*p* = 0.009). Maximum constriction (Figure 3C) did not differ significantly between the groups. These data infer that insulin treatment of GDM patients changes the vasoreactivity of their omental arteries when exposed to ex vivo ET-1.

### 3.3. ET-1 Antagonists, BQ123 and BQ788, Inhibit ET-1 Induced Constriction in Omental Arteries from Healthy Pregnancies and Those Complicated by GDM

**Figure 4:**
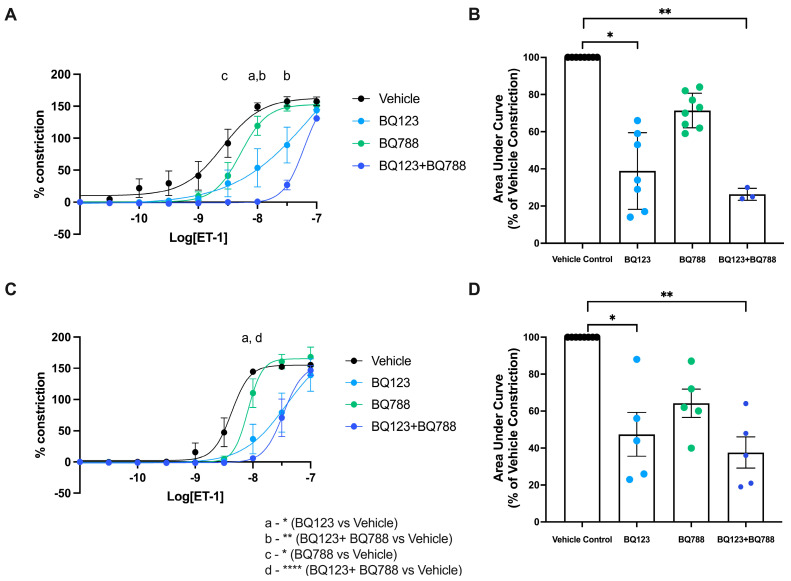
Antagonism of the ET−1 receptors alters ET−1-induced vasoconstriction of omental arteries from pregnancy. Omental arteries were collected from healthy-term pregnancies (**A**,**B**), or those complicated by GDM with insulin management as treatment (**C**,**D**). Prior to constriction with recombinant ET−1, arteries were incubated with ET_A_ antagonist, BQ123 (1 µM, blue circles), and/or ET_B_ antagonist, BQ788 (5 µM, green circles indicate BQ788 alone and purple circles indicate a combination), or vehicle control (black circles). (**A**,**C**) Constriction curves were generated by increasing logarithmic doses of ET−1, and the percent of constriction was normalized to a maximum constriction induced by 50 mM of potassium salt solution (KPSS). (**B**,**D**) The area under the curve analysis is expressed as a percentage of the vehicle treatment constriction. Individual points represent individual patients. The data is expressed as mean ± SEM. Significance is indicated as follows: * *p* < 0.05, ** *p* < 0.01 and **** *p* < 0.0001.

Given we observed a decreased constrictory response to ET-1 in vessels collected from GDM pregnancies treated with insulin, we next set out to investigate whether differences in levels of ET-1 receptors might be responsible for this altered response. We observed that both antagonists, BQ123 and BQ788, singularly and in combination, were successful in dampening constriction induced by ET-1 in healthy pregnant arteries. At dose 10^−8.5^ M (BQ788 vs. vehicle, *p* = 0.034), 10^−8^ M (BQ123, *p* = 0.038, and BQ123 and BQ788, *p* = 0.003) and 10^−7.5^ M (BQ123 and BQ788, *p* = 0.007) of ET-1, the constriction produced with the antagonists respectively was significantly less than the vehicle (Figure 4A). Therefore, the area under the curve (Figure 4B) also indicates decreased total constriction following pre-treatments with antagonist BQ123 (*p* = 0.035) and BQ123 + BQ788 (*p* = 0.008) compared to vehicle control. In contrast, only the antagonistic actions of BQ123 alone (*p* = 0.014) and BQ123 + BQ788 (*p* < 0.0001) were successful in decreasing ET-1 induced constriction at dose 10^−8^ M in omental arteries collected from GDM pregnancies treated with insulin (Figure 4C). The area under the curve relative to vehicle control was significantly decreased with BQ123 (*p* = 0.031) and BQ123 + BQ788 (*p* = 0.002) pre-treatment (Figure 4D).

### 3.4. The mRNA Expression of ET_A_, ET_B_, VCAM, and ICAM in Maternal Omental Arteries Is Not Altered in GDM

**Figure 5:**
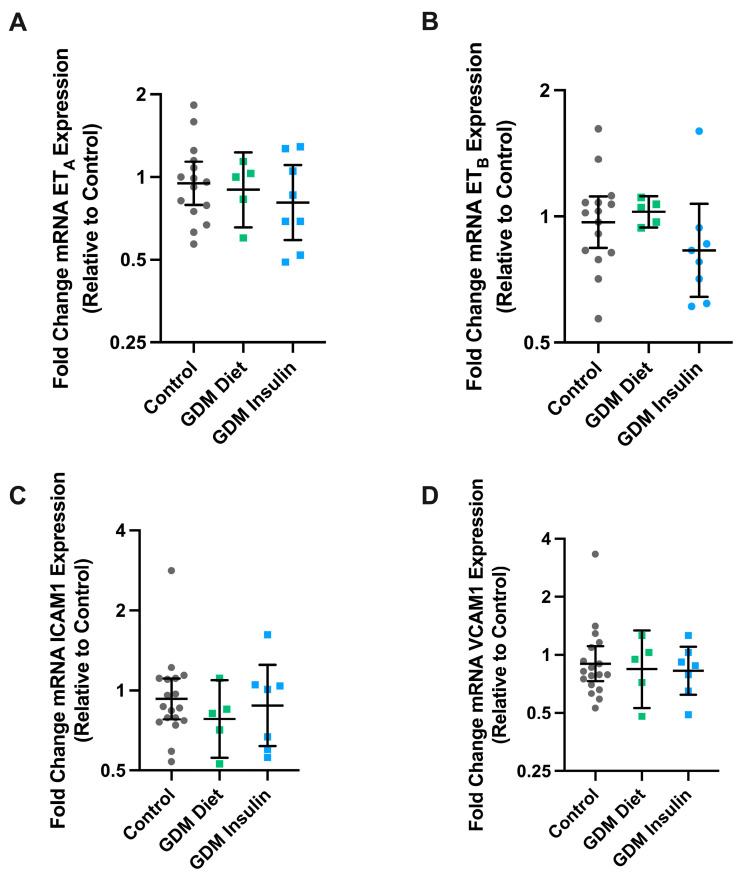
The mRNA expression of endothelin receptors and markers of endothelial dysfunction were not altered in omental arteries collected from GDM pregnancies. ET-1 receptors ET_A_ (**A**), ET_B_ (**B**), and markers of endothelial dysfunction, intercellular adhesion molecule-1 (ICAM1; (**C**)) and vascular cell adhesion molecule-1 (VCAM1; (**D**)), were examined by qPCR in omental vessels collected from pregnancies complicated by GDM managed with diet intervention (*n* = 5, green squares) or insulin treatment (*n* = 8, blue squares), compared to healthy gestation-matched controls (*n* = 15, grey circles). Data is expressed as the log fold change of 2^−ΔΔCT^ Geomean (95% CI) relative to normal control.

We next set out to determine whether the differences in response to ET-1 in pregnancies complicated by GDM (managed with insulin treatment) might be due to changes in the expression of the ET- receptors. Expression of ET_A_ and ET_B_ mRNA was not altered in omental arteries from pregnancies complicated by GDM treated with insulin or diet intervention, compared to healthy gestation-matched controls (Figure 5). As GDM is associated with endothelial dysfunction, we also assessed whether GDM might alter the expression of endothelial dysfunction markers ICAM1 and VCAM1. However, there were no significant differences observed in the mRNA expression of VCAM1 or ICAM1 in arteries collected from pregnancies complicated by GDM with either diet or insulin intervention.

### 3.5. ET-1 Induced Constriction of Omental Arteries Is Reduced by Exposure to Insulin

**Figure 6:**
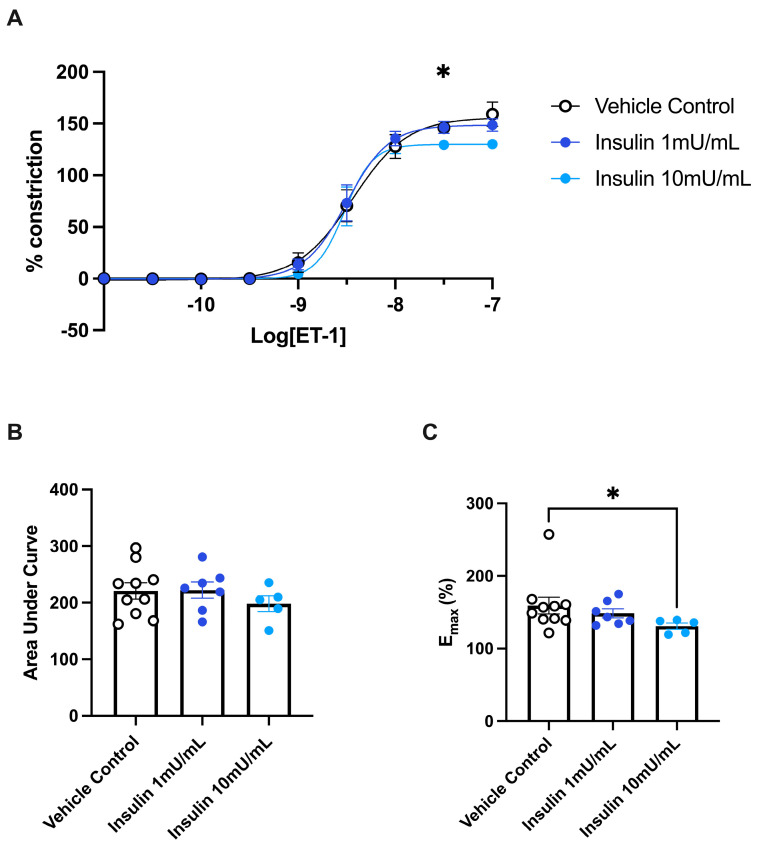
Insulin treatment decreases the maximum constriction induced by ET−1 in healthy maternal omental arteries. Constriction dose-response curves (**A**), analysis of the area under the curve (**B**), and maximum constriction (**C**) generated by increasing logarithmic doses of ET-1 in omental arteries collected from healthy pregnancies (term) incubated with 1 mU/mL insulin (*n* = 7, purple circles) or 10 mU/mL insulin (*n* = 5, blue circles), compared to vehicle control (*n* = 12, white circles) for 30 min. The percent constriction was normalised to the maximum constriction induced by 50 mM potassium salt solution (KPSS). Individual points represent artery reactivity from individual patients. The data is expressed as mean ± SEM. Significance is as follows; * *p* < 0.05 (control vs. insulin 10 mU/mL).

To investigate whether the difference in ET-1-induced constriction in GDM insulin-treated patients was due to the use of insulin as a treatment type, we next investigated the effect of exposing healthy pregnant arteries to insulin on ET-1-driven constriction. There were no significant changes in the constriction induced by ET-1 in omental arteries incubated with 1 mU/mL insulin (Figure 6). However, in arteries incubated with 10 mU/mL insulin we observed reduced vascular constriction to ET-1 compared to vehicle control. At dose 10^−7.5^ (insulin 10 mU/mL compared to control, *p* = 0.029) of ET-1, the constriction induced was significantly less (Figure 6A). The maximum constriction induced by ET-1 following incubation in 10 mU/mL insulin was significantly less than the vehicle control (*p* = 0.013; Figure 6C). However, the analysis of the area under the curve revealed that there was no significant difference in ET-1-induced constriction between any of the treatment groups (Figure 6B). Taken together, this infers that treatment of omental arteries with 10 mU/mL of insulin for a short period of time (30 min) can alter the vasoreactivity of maternal resistance arteries to ET-1 in an ex vivo setting.

### 3.6. Models of Endothelial Dysfunction and Treatment with Insulin

We next set out to further interrogate whether insulin treatment may be reducing endothelial dysfunction directly. Here, we examined whether treatment with ET-1 antagonists BQ123/BQ788 or insulin (1 mU/mL and 10 mU/mL doses (Supplementary Figure S1) can reduce markers associated with endothelial dysfunction ET-1, VCAM1 and ICAM1. Tumor necrosis factor (TNF-α) was used to induce endothelial dysfunction. This dose of TNF-α increased expression of VCAM1 in endothelial cells treated with TNF-α (*p* = 0.0079) but not ET-1 or ICAM1 (Supplementary Figure S1). Neither of the ET-1 antagonists nor insulin treatment (1 or 10 mU/mL) affected expression of ET-1 (Supplementary Figure S1A–C), VCAM1 (Supplementary Figure S1D–F), or ICAM1 (Supplementary Figure S1G–I).

### 3.7. Conditioned Media Obtained from Placental Tissue Exposed to High Glucose Does Not Alter Omental Artery Vasoconstriction to ET-1

Finally, we investigated whether conditioned media obtained from placental tissue explants exposed to high or standard glucose levels altered the vasoreactivity of omental arteries when constricted with ET-1. Firstly, to control for the higher glucose levels in the media, we examined whether non-conditioned media altered omental vasoreactivity to ET-1. We observed no difference between omental arteries exposed to high (25 M) glucose or standard (5 M) glucose media (Figure 5A). Secondly, the vasoconstriction response induced by ET-1 was not significantly altered by pre-incubation of omental arteries in placental explant conditioned media containing high (25 M) glucose compared to placental explant conditioned media containing standard (5 M) glucose (Supplementary Figure S2A). Overall, similar constriction profiles were observed in all omental artery pre-treatment experimental conditions; this is further demonstrated in the total constriction analysis (area under the curve; Supplementary Figure S2B) and the maximum constriction reached with ET-1 (Supplementary Figure S2C).

## 4. Discussion

Despite a known association between gestational diabetes and endothelial dysfunction [24], relatively few studies have investigated the potential role of ET-1 in GDM. In particular, the action of ET-1 on endothelial and vascular response in GDM, given the key role that ET-1 plays in regulating vascular tone. Here, we interrogated the potential contribution of ET-1 to the vascular impairment associated with GDM. Additionally, we explored the ET-1 pathway in GDM pregnancies treated with either diet or insulin management; when diet intervention alone cannot reduce hyperglycemia, insulin treatment is administered. Overall, there was no clear difference in the circulating levels or vascular expression of ET-1 in GDM, with either management via diet intervention or insulin treatment. Further, maternal arteries did not constrict differently in response to ET-1 with or without disease. However, in pregnancies complicated with GDM, managed with insulin, we observed a significant decrease in ET-1-induced constriction of the maternal arteries; these actions may be driven by the direct effects of insulin on the maternal vasculature.

Endothelial dysfunction is thought to play a role in the pathophysiology that underpins GDM. Markers of endothelial dysfunction may provide more insight into the role of endothelial impairment driving the disease stages; markers include VCAM1, ICAM1, and ET-1 [25,26,27,28]. Several studies have reported increases in circulating ET-1 with GDM [17,29,30], however, these studies included cohorts where there was concurrent development of preeclampsia or gestational hypertension. Given an increase in circulating ET-1 is also strongly associated with hypertensive disorders of pregnancy [31,32,33], the inclusion of such samples is an important confounder. Simultaneous decreases in circulating nitric oxide (a key molecule essential for vasodilation) have been correlated with increased ET-1 levels [29]. Studies that exclude hypertensive disorders of pregnancy (including our study) have not demonstrated significant differences in circulating ET-1 levels with GDM [34]. Additionally, when further stratifying samples by the clinical management of GDM, we observed no difference in plasma levels of ET-1 in pregnancies complicated by GDM managed by diet or insulin intervention. This suggests that ET-1 specifically may only be altered with clinical manifestations of vascular dysfunction (for example, hypertension); however, the physiological balance of key vasoconstrictors and vasodilators may still be altered in GDM [35], and this warrants further investigation.

Studies to date have predominantly explored the association of GDM with impaired endothelium-dependent vasodilation [28,36,37]. Subcutaneous arteries collected from pregnancies complicated by GDM have exhibited poor relaxation in response to acetylcholine [36], and furthermore, myometrial arteries have similarly shown impaired bradykinin vasodilation [37]. Of note, both acetylcholine and bradykinin are endothelium-dependent vasodilators. Within these studies, patients who were treated with either insulin or diet intervention were grouped together. The effect of GDM on systemic vasoconstriction (particularly driven by the outer vascular smooth muscle layer, as demonstrated in Figure 1) has not been widely studied. In the current study, we demonstrated that ET-1-induced constriction in omental arteries from patients whose pregnancies were complicated by GDM was altered only in patients treated with insulin; those who were treated with insulin demonstrated a decrease in the constriction induced by ET-1 ex vivo. The constriction of arteries collected from patients who were being managed by dietary intervention was not significantly different from healthy controls.

Although there was a clear decrease in the ET-1-induced constriction in omental arteries from GDM pregnancies treated with insulin, we did not observe a difference in the mRNA expression of ET_A_ and ET_B_ receptors in the maternal vasculature between groups. To further uncover the mechanisms behind this change in vasoconstriction, we next investigated the function of ET-1 receptors using specific antagonists to ET_A_ and ET_B_, enabling the examination of their functional relevance.

The ET_A_ receptor antagonist, BQ123, was effective in dampening ET-1-induced constriction in omental arteries from both healthy pregnancies and those complicated by GDM treated with insulin. Conversely, inhibition of ET-1 induced constriction with the specific ET_B_ receptor antagonist, BQ788, was observed in arteries from healthy pregnancies but not in arteries collected from pregnancies complicated with GDM treated with insulin. Given we did not observe a change in expression of endothelin receptors in the vasculature of pregnancies managed with insulin treatment, we speculate that the distribution of ET_B_ receptors may be altered by insulin treatment (from the vascular smooth muscle to the endothelium) and hence their ability to cause constriction is lost. Further investigations into the spatiotemporal distribution of endothelin receptors in maternal vasculature following insulin treatment are required.

Insulin treatment is administered for the management of GDM when diet intervention alone cannot control blood glucose levels. Hence, the importance in the current study to stratify these two groups to allow us to distinguish whether it was the severity of the disease in this cohort that gave rise to the differences in vascular response to ET-1, or a direct effect of insulin on the vasculature. Typically, insulin is thought to increase nitric oxide availability and hence act as a nitric oxide-dependent dilator of resistance vessels. Conversely, elevated levels of insulin can simultaneously induce vasoconstriction mediated by endothelin and reactive oxygen free radicals [38]. Though there is a correlation between insulin resistance and increased ET-1 activity [39], insulin can inhibit ET-1 release from mouse microvascular endothelial cells [40]. Thus, it was imperative to demonstrate the altered vascular response with insulin treatment in vivo and ex vivo. We observed that treatment of arteries ex vivo with insulin reduced ET-1-induced constriction.

Investigating whether the vasculature itself is altered in GDM cases where diet alone cannot control glucose is difficult due to limitations in patient samples, whereby patients’ blood sugar levels must be immediately managed with insulin to mitigate potential impacts to fetal growth. Hence, we modeled a featured GDM-like hyperglycemic environment. Hyperglycemia can have profound impacts on endothelial behavior, specifically impairing HUVEC proliferation, migration, and tube formation, and subsequently, vascular function may be altered [41]. High levels of glucose increase nitric oxide synthesis via eNOS in HUVECs [42]. Previous studies have shown that hyperglycemia is associated with decreased flow-mediated vasodilation [43] and an increase in the constrictory response to the smooth muscle-dependent thromboxane agonist, U46619, in a placental perfusion model [44]. Further, hyperglycemia has also augmented the vascular response to ET-1 in human and porcine retinal vessels following incubation [45]. We did not observe differences in the constrictory response to ET-1 with increased glucose availability in omental arteries (collected from pregnancy; Supplementary Figure S2), suggesting that hyperglycemia in GDM is unlikely to be responsible for the observed altered response. Whilst our aim was to investigate immediate effects on vasoreactivity, further studies examining whether longer exposures to high glucose levels might alter the distribution of endothelin receptors are warranted.

Indeed, we found that insulin itself is more likely to be responsible for the reduced ET-1-induced constriction. Here we demonstrate that short-term exposure of even healthy pregnant omental arteries to insulin (30 min) can decrease ET-1-induced constriction, highlighting its potential protective effects on maternal vasculature. Future studies may investigate whether prolonged and repeated exposure to insulin ex vivo, modeling the daily insulin injections in patients with GDM, may have further effects. In concurrence with our findings, exposing normotensive, nonpregnant subcutaneous arteries to exogenous insulin (10 mU/mL) has previously decreased the constriction induced by noradrenaline in a dose-dependent manner [41]. Noradrenaline directly induces constriction of the vascular smooth muscle, and hence, insulin may have a direct effect on the vascular smooth muscle rather than the endothelium.

There are potential benefits of insulin restoring adenosine transport and, hence, endothelial function in GDM [42,43]. However, in the current study, we observed no changes in the expression of *ET-1*, *VCAM1,* or *ICAM1* in TNFα induced endothelial dysfunction in HUVECs when treated with 10 mU/mL of insulin for 24 h (Supplementary Figure S1). Previously, insulin treatment did not reverse GDM associated changes in HUVECs [46]. This further indicates that insulin may not influence the endothelium specifically.

A key strength of this study is the use of primary human gestational samples, including plasma, placenta, omental arteries, and isolated primary endothelial cells, obtained from pregnancies complicated by GDM and/or healthy controls. Most importantly, in this study, our patient characteristics were defined, allowing the important stratification of the GDM cohort by clinical management (diet or insulin treated). As this study demonstrates that insulin can directly reduce vasoconstriction, we suggest that in future assessments of other therapies to treat GDM, researchers should consider whether pregnancies given insulin treatment are appropriate to include in a control group, given it might mask direct vascular effects.

Collectively, these results infer that insulin treatment directly acts on the vasculature to reduce ET-1-induced vasoconstriction. We hypothesize that this could be driven by changes in the distribution of constrictory ET_B_ receptors on the vascular smooth muscle. This new knowledge is important to the field, particularly in the differences we have detected in vascular response between samples from GDM pregnancies managed with dietary changes versus insulin. Further research could determine whether this could be relevant in choosing the intervention provided to GDM pregnancies, considering their increased risk of vascular impairment. Given the rise in pregnancies complicated by GDM, it is vital that an improved understanding of the mechanisms driving this complication is elucidated to allow for better development of therapeutic strategies to improve pregnancy outcomes and long-term maternal health.

## Figures and Tables

**Table 1:** Maternal clinical characteristics of plasma samples from pregnancies complicated by gestational diabetes mellitus (GDM), managed with diet intervention or insulin treatment and healthy controls.

	Control	GDM Diet	*p* Value	GDM Insulin	*p* Value
*n*	42	12		13	
Body Mass Index (BMI) (median [IQR])	25.86 [22.45, 29.38]	29.41 [25.05, 32.15]	0.120	31.23 [25.3, 38.3]	0.009
Gestation at delivery (median [IQR])	39.59 [39.00, 40.43]	37.70 [38.04, 38.93]	<0.001	38.54 [38.14, 39]	0.049
Highest Systolic Blood Pressure during admission (mmHg) (median [IQR])	118.21 [110.00, 120.00]	120.83 [113.75, 126.25]	0.594	121.92 [115.00, 125.00]	0.339
Highest Diastolic Blood Pressure during admission (mmHg) (median [IQR])	72.21 [70.00, 78.75]	76.25 [70.00, 80.00]	0.187	75.77 [70.00, 80.00]	0.247
Birthweight (mean (SD))	3474.62 (661.33)	3361.92 (724.49)	0.829	3807.85 (451)	0.196
Parity (%)			0.019		0.308
0	14 (33.3)	4 (33.4)		3 (23.1)	
1	24 (57.1)	6 (50)		4 (30.8)	
2	4 (9.6)	1 (8.3)		5 (38.4)	
3	0 (0)	1 (8.3)		1 (7.7)	
Fetal sex (%)			0.513		>0.999
Female	18 (32.9)	7 (58.3)		5 (38.5)	
Male	24 (57.1)	5 (41.7)		8 (61.5)	

**Table 2:** Maternal clinical characteristics of omental samples from pregnancies complicated by gestational diabetes mellitus (GDM) and managed with diet intervention or insulin treatment and healthy controls.

	Control	GDM Diet	*p* Value	GDM Insulin	*p* Value
*n*	36	9		21	
Body Mass Index (BMI) (median [IQR])	23.38 [21.79, 29.48]	29.29 [27.35, 32.56]	0.025	27.54 [26.25, 32.89]	0.019
Gestation at delivery (median [IQR])	39.00 [38.50, 39.10]	38.60 [38.50, 39.00]	0.605	38.60 [38.20, 39.00]	0.046
Highest Systolic Blood Pressure during admission (mmHg) (median [IQR])	120.00 [120.00, 130.00]	120.00 [120.00, 125.00]	0.965	125.00 [120.00, 130.00]	0.125
Highest Diastolic Blood Pressure during admission (mmHg) (median [IQR])	75.00 [70.00, 80.00]	75.00 [70.00, 75.00]	0.895	75.00 [75.00, 80.00]	0.198
Birthweight (mean (SD))	3527.17 (407.89)	3676.67 (271.11)	0.305	3681.57 (683.76)	0.289
Parity (%)			0.154		0.512
1	5 (14.3)	0 (0.0)		2 (9.5)	
2	21 (60.0)	6 (66.7)		11 (52.4)	
3	9 (25.7)	2 (22.2)		7 (33.3)	
4	0 (0.0)	1 (11.1)		1 (4.8)	
Fasting Glucose (median (SD))	4.40 (0.38)	4.90 (0.58)	0.005	5.20 (0.48)	<0.001
Glucose Tolerance Test; 1 h (mean (SD))	7.19 (1.26)	10.52 (1.12)	<0.001	10.47 (2.22)	<0.001
Glucose Tolerance Test; 2 h (mean (SD))	5.98 (1.13)	7.87 (2.41)	0.001	8.18 (2.14)	<0.001
Fetal sex (%)			0.941		0.654
Female	19 (52.8)	4 (44.4)		9 (42.9)	
Male	17 (47.2)	5 (55.6)		12 (57.1)	

## Data Availability

Data available upon reasonable request from the corresponding author.

## Supplementary Materials

The following supporting information can be downloaded at: https://www.mdpi.com/article/10.3390/biomedicines11102660/s1, Supplementary Figure S1: Treatment of HUVECs with insulin did not alter TNFα induced markers of endothelial dysfunction; Supplementary Figure S2: ET-1 constriction is not altered by placental conditioned media at standard and high glucose concentrations.

## References

[1] Plows J.F., Stanley J.L., Baker P.N., Reynolds C.M., Vickers M.H. (2018). The Pathophysiology of Gestational Diabetes Mellitus. Int. J. Mol. Sci..

[2] Zhu Y., Zhang C. (2016). Prevalence of Gestational Diabetes and Risk of Progression to Type 2 Diabetes: A Global Perspective. Curr. Diabetes Rep..

[3] Saravanan P., Magee L.A., Banerjee A., Coleman M.A., Von Dadelszen P., Denison F., Farmer A., Finer S., Fox-Rushby J., Holt R. (2020). Gestational diabetes: Opportunities for improving maternal and child health. Lancet Diabetes Endocrinol..

[4] Garrison A. (2015). Screening, diagnosis, and management of gestational diabetes mellitus. Am. Fam. Physician.

[5] Fu J., Retnakaran R. (2022). The life course perspective of gestational diabetes: An opportunity for the prevention of diabetes and heart disease in women. EClinicalMedicine.

[6] McKenzie-Sampson S., Paradis G., Healy-Profitós J., St-Pierre F., Auger N. (2018). Gestational diabetes and risk of cardiovascular disease up to 25 years after pregnancy: A retrospective cohort study. Acta Diabetol..

[7] Carpenter M.W. (2007). Gestational Diabetes, Pregnancy Hypertension, and Late Vascular Disease. Diabetes Care.

[8] Kornacki J., Gutaj P., Kalantarova A., Sibiak R., Jankowski M., Wender-Ozegowska E. (2021). Endothelial Dysfunction in Pregnancy Complications. Biomedicines.

[9] Hynynen M.M., Khalil R.A. (2006). The vascular endothelin system in hypertension--recent patents and discoveries. Recent. Pat. Cardiovasc. Drug Discov..

[10] Arai H., Hori S., Aramori I., Ohkubo H., Nakanishi S. (1990). Cloning and expression of a cDNA encoding an endothelin receptor. Nature.

[11] Clozel M., Gray G.A., Breu V., Löffler B.M., Osterwalder R. (1992). The endothelin ETB receptor mediates both vasodilation and vasoconstriction in vivo. Biochem. Biophys. Res. Commun..

[12] Khalil R.A. (2011). Modulators of the vascular endothelin receptor in blood pressure regulation and hypertension. Curr. Mol. Pharmacol..

[13] Fukuroda T., Ozaki S., Ihara M., Ishikawa K., Yano M., Nishikibe M. (1994). Synergistic inhibition by BQ-123 and BQ-788 of endothelin-1-induced contractions of the rabbit pulmonary artery. Br. J. Pharmacol..

[14] Kowalczyk A., Kleniewska P., Kolodziejczyk M., Skibska B., Goraca A. (2015). The role of endothelin-1 and endothelin receptor antagonists in inflammatory response and sepsis. Arch. Immunol. Ther. Exp..

[15] Pantham P., Aye I.L., Powell T.L. (2015). Inflammation in maternal obesity and gestational diabetes mellitus. Placenta.

[16] Lygnos M.C., Pappa K.I., Papadaki H.A., Relakis C., Koumantakis E., Anagnou N.P., Eliopoulos G.D. (2006). Changes in maternal plasma levels of VEGF, bFGF, TGF-beta1, ET-1 and sKL during uncomplicated pregnancy, hypertensive pregnancy and gestational diabetes. In Vivo.

[17] Wolff K., Carlstrom K., Fyhrquist F., Hemsén A., Lunell N.O., Nisell H. (1997). Plasma endothelin in normal and diabetic pregnancy. Diabetes Care.

[18] Telejko B., Zonenberg A., Kuzmicki M., Modzelewska A., Niedziolko-Bagniuk K., Ponurkiewicz A., Nikolajuk A., Gorska M. (2009). Circulating asymmetric dimethylarginine, endothelin-1 and cell adhesion molecules in women with gestational diabetes. Acta Diabetol..

[19] Hoffman L., Nolan C., Wilson J.D., Oats J.J., Simmons D. (1998). Gestational diabetes mellitus—Management guidelines. The Australasian Diabetes in Pregnancy Society. Med. J. Aust..

[20] Fato B.R., de Alwis N., Beard S., Binder N.K., Pritchard N., Tong S., Kaitu’u-Lino T.J., Hannan N.J. (2022). Serum Collected from Preeclamptic Pregnancies Drives Vasoconstriction of Human Omental Arteries-A Novel Ex Vivo Model of Preeclampsia for Therapeutic Development. Int. J. Mol. Sci..

[21] de Alwis N., Fato B.R., Beard S., Binder N.K., Kaitu’u-Lino T.u.J., Onda K., Hannan N.J. (2022). Assessment of the Proton Pump Inhibitor, Esomeprazole Magnesium Hydrate and Trihydrate, on Pathophysiological Markers of Preeclampsia in Preclinical Human Models of Disease. Int. J. Mol. Sci..

[22] Onda K., Tong S., Beard S., Binder N., Muto M., Senadheera S.N., Parry L., Dilworth M., Renshall L., Brownfoot F. (2017). Proton Pump Inhibitors Decrease Soluble fms-Like Tyrosine Kinase-1 and Soluble Endoglin Secretion, Decrease Hypertension, and Rescue Endothelial Dysfunction. Hypertension.

[23] Tong S., Kaitu’u-Lino T.U.J., Onda K., Beard S., Hastie R., Binder N.K., Cluver C., Tuohey L., Whitehead C., Brownfoot F. (2015). Heme Oxygenase-1 Is Not Decreased in Preeclamptic Placenta and Does Not Negatively Regulate Placental Soluble fms-Like Tyrosine Kinase-1 or Soluble Endoglin Secretion. Hypertension.

[24] Sena C.M., Pereira A.M., Seiça R. (2013). Endothelial dysfunction—A major mediator of diabetic vascular disease. Biochim. Biophys. Acta (BBA) Mol. Basis Dis..

[25] Göbl C.S., Bozkurt L., Yarragudi R., Prikoszovich T., Tura A., Pacini G., Koppensteiner R., Kautzky-Willer A. (2014). Biomarkers of endothelial dysfunction in relation to impaired carbohydrate metabolism following pregnancy with gestational diabetes mellitus. Cardiovasc. Diabetol..

[26] Mordwinkin N.M., Ouzounian J.G., Yedigarova L., Montoro M.N., Louie S.G., Rodgers K.E. (2013). Alteration of endothelial function markers in women with gestational diabetes and their fetuses. J. Matern. Fetal Neonatal Med..

[27] Siddiqui K., George T.P., Nawaz S.S., Joy S.S. (2019). VCAM-1, ICAM-1 and selectins in gestational diabetes mellitus and the risk for vascular disorders. Future Cardiol..

[28] Gungor O., Gazi E., Ozkececi G., Cakir Gungor A.N., Cevizci S., Hacivelioglu S., Temiz A., Mert N., Koken G. (2015). Is abnormal glucose metabolism during pregnancy related to endothelial dysfunction?. J. Matern. Fetal Neonatal Med..

[29] Lan Q., Zhou Y., Zhang J., Qi L., Dong Y., Zhou H., Li Y. (2022). Vascular endothelial dysfunction in gestational diabetes mellitus. Steroids.

[30] Al-Ofi E., Alrafiah A., Maidi S., Almaghrabi S., Hakami N. (2021). Altered Expression of Angiogenic Biomarkers in Pregnancy Associated with Gestational Diabetes. Int. J. Gen. Med..

[31] LaMarca B.B.D., Cockrell K., Sullivan E., Bennett W., Granger J.P. (2005). Role of Endothelin in Mediating Tumor Necrosis Factor-Induced Hypertension in Pregnant Rats. Hypertension.

[32] Baksu B., Davas I., Baksu A., Akyol A., Gulbaba G. (2005). Plasma nitric oxide, endothelin-1 and urinary nitric oxide and cyclic guanosine monophosphate levels in hypertensive pregnant women. Int. J. Gynecol. Obstet..

[33] Lu Y.-P., Hasan A.A., Zeng S., Hocher B. (2017). Plasma ET-1 Concentrations Are Elevated in Pregnant Women with Hypertension -Meta-Analysis of Clinical Studies. Kidney Blood Press. Res..

[34] Lappas M. (2014). Markers of endothelial cell dysfunction are increased in human omental adipose tissue from women with pre-existing maternal obesity and gestational diabetes. Metabolism.

[35] Swiderski S., Celewicz Z., Miazgowski T., Ogonowski J. (2010). Maternal endothelin-1 and cyclic guanosine monophosphate concentrations in pregnancies complicated by pregravid and gestational diabetes mellitus. Gynecol. Obstet. Investig..

[36] Knock G.A., McCarthy A.L., Lowy C., Poston L. (1997). Association of gestational diabetes with abnormal maternal vascular endothelial function. Br. J. Obstet. Gynaecol..

[37] Chirayath H.H., Wareing M., Taggart M.J., Baker P.N. (2010). Endothelial dysfunction in myometrial arteries of women with gestational diabetes. Diabetes Res. Clin. Pract..

[38] Markos F., Shortt C.M., Edge D., Ruane-O’Hora T., Noble M.I. (2014). Immediate direct peripheral vasoconstriction in response to hyperinsulinemia and metformin in the anesthetized pig. Physiol. Res..

[39] Kalani M. (2008). The importance of endothelin-1 for microvascular dysfunction in diabetes. Vasc. Health Risk Manag..

[40] Worm J. (2014). Insulin and insulin-like growth factor-1 inhibit release of endothelin-1 from mouse micro-vascular endothelial cells. Atherosclerosis.

[41] McNally P.G., Lawrence I.G., Watt P.A., Hillier C., Burden A.C., Thurston H. (1995). The effect of insulin on the vascular reactivity of isolated resistance arteries taken from healthy volunteers. Diabetologia.

[42] Muñoz G., Martín R.S., Farías M., Cea L., Vecchiola A., Casanello P., Sobrevia L. (2006). Insulin restores glucose inhibition of adenosine transport by increasing the expression and activity of the equilibrative nucleoside transporter 2 in human umbilical vein endothelium. J. Cell. Physiol..

[43] Westermeier F., Salomón C., González M., Puebla C., Guzmán-Gutiérrez E., Cifuentes F., Leiva A., Casanello P., Sobrevia L. (2011). Insulin restores gestational diabetes mellitus-reduced adenosine transport involving differential expression of insulin receptor isoforms in human umbilical vein endothelium. Diabetes.

[44] Reed L.C., Estrada S.M., Walton R.B., Napolitano P.G., Ieronimakis N. (2018). Evaluating maternal hyperglycemic exposure and fetal placental arterial dysfunction in a dual cotyledon, dual perfusion model. Placenta.

[45] Chen Y.-L., Rosa R.H., Kuo L., Hein T.W. (2020). Hyperglycemia Augments Endothelin-1–Induced Constriction of Human Retinal Venules. Transl. Vis. Sci. Technol..

[46] Zhou J., Ni X., Huang X., Yao J., He Q., Wang K., Duan T. (2016). Potential Role of Hyperglycemia in Fetoplacental Endothelial Dysfunction in Gestational Diabetes Mellitus. Cell Physiol. Biochem..

