# Supplementary material for: The Regulation of Endothelin-1 in Pregnancies Complicated by Gestational Diabetes: Uncovering the Vascular Effects of Insulin
Source: Biomedicines. 2023 Sep 28;11(10):2660. doi: 10.3390/biomedicines11102660 (PMC10603897; doi:10.3390/biomedicines11102660)
Supplement: Supplementary file 1 [file biomedicines-11-02660-s001.zip › biomedicines-2578967-supplementary.pdf]

The Regulation of Endothelin-1 in Pregnancies Complicated by Gestational Diabetes: Uncovering the Vascular Effects of Insulin

Bianca R. Fato, Sally Beard, Natalie K. Binder, Natasha Pritchard, Tu'uhevaha J. Kaitu'u-Lino, Natasha de Alwis and Natalie J. Hannan

Supplementary Material

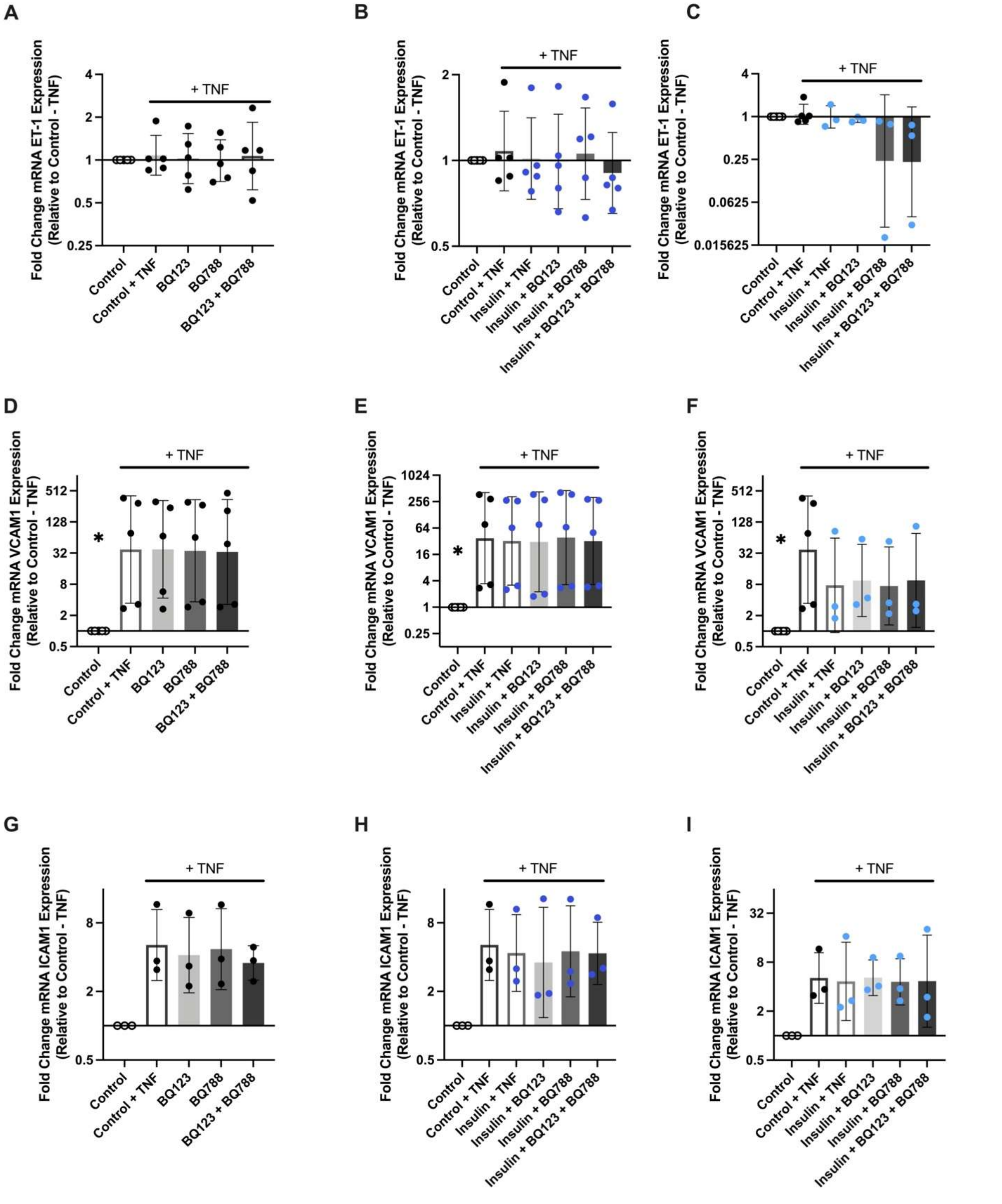

Supplementary Figure S1. Treatment of HUVECs with insulin did not alter TNFα induced markers of endothelial dysfunction. Expression of *ET-1* (A-C) , *VCAM1* (D-F), and *ICAM1* (G-I) mRNA was not altered in HUVECs treated with insulin (1mU/mL; B, E, H) or 10mU/mL; C, F, I)) or receptor antagonists BQ123 and BQ788 (A, D, G) for 24 hours, following induction of endothelial dysfunction via treatment with 1ng/mL TNFα. Individual points represent individual patients (n=3-5), data is expressed as the log fold change of ΔΔCT Geomean (with geometric SD), relative to control treatment. Significance is as follows: * $p<0.01$ (compared to Control +TNF).

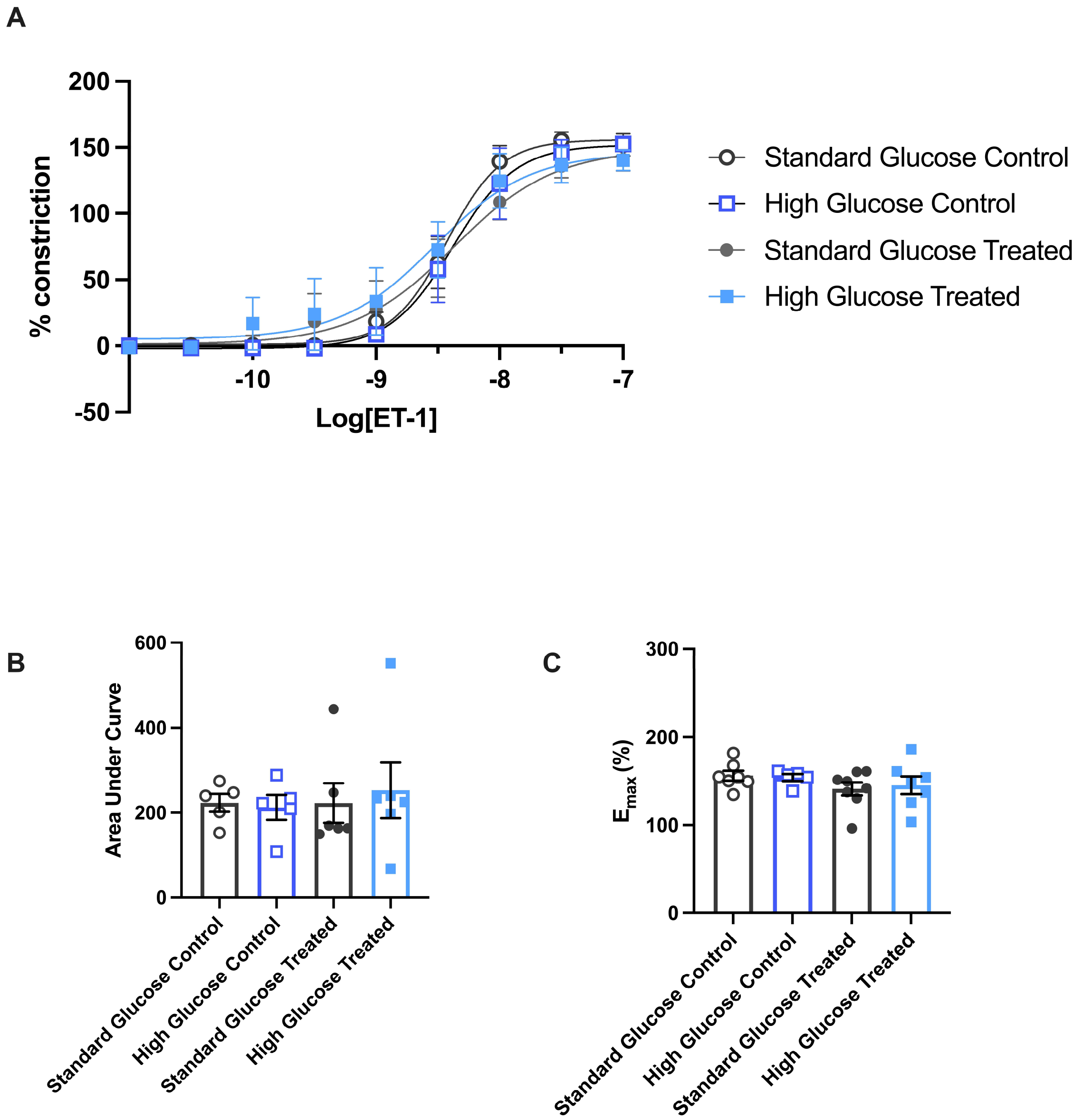

Supplementary Figure S2. ET-1 constriction is not altered by placental conditioned media at standard and high glucose concentrations. Constriction curves (A), analysis of the area under the curve (B) and maximum constriction (C) generated by increasing logarithmic doses of a known vasoconstrictor, ET-1, in omental arteries collected from uncomplicated pregnancies. The percentage of constriction was normalised to the maximum constriction induced by 50mM potassium salt solution (KPSS). Individual points represent individual patients (n=7). The data is expressed as mean ± SEM.
